# Identification of household dangers by parents from adult versus child visual perspective

**DOI:** 10.5249/jivr.vo113i2.1654

**Published:** 2021-07

**Authors:** Jackson Vane, Lynne Fullerton, Robert Sapién

**Affiliations:** ^a^ Department of Emergency Medicine, University of New Mexico Health Sciences Center, Albuquerque, NM, USA.; ^*b*^ Rady Children’s Hospital/UC San Diego School of Medicine, San Diego, CA, USA.

**Keywords:** Household safety, Hazard identification, Childproofing

## Abstract

**Background:**

This study utilized videos from a child’s and an adult’s perspective to determine whether perspective influences the number of hazards identified by parents.

**Methods:**

The study measured number of household dangers parents’ identified. Parents (n=106) were randomized to view either the child or adult perspective videos. Groups did not differ with respect to median age (p=0.51), education (p=0.55), or number of children living at home (p=0.64).

**Results:**

Median number of hazards identified in the bedroom was 3 for participants watching videos taken at either adult or child perspective (p=0.32). Parents viewing child perspective videos of the kitchen identified significantly more hazards (median=4) than parents viewing adult perspective videos (median=3) (p=0.0001).

**Conclusions:**

Although video height (perspective) did not influence the number of hazards identified in the bedroom, parents who observed the kitchen video taken at a child’s height identified more hazards than those viewing a video at adult height.

## Introduction

In 2016, nearly 7,000 U.S. children ages 18 years and younger died from unintentional injuries.^1^ Unintentional injury is the leading cause of death for children 1-18 years old.^2^ In 2015, there were 8.2 million pediatric emergency department (ED) visits to treat injury and poisoning.^3^ Leading causes for pediatric ED unintentional injury visits in 2015 include falls (2.4 million), struck by/against (1.6 million), and overexertion (0.7 million).^4^

The Centers for Disease Control (CDC), through their National Action Plan for Child Injury Prevention, suggested that most injuries are preventable and further education about injury prevention may help families safeguard their homes and reduce childhood injury.^5^ In 2012, a Cochrane review was performed compiling 98 studies to evaluate whether a home safety educational intervention would influence the practice of making a home safer. Multiple studies and tactics were evaluated, including in-home education, providing goods, and pamphlets. One such study utilizing pamphlets, showed that there was poor recall after several weeks, even when using information appropriate for people with limited reading skills. Kendrick et al, who performed the Cochrane review, concluded that there was no consistent method that ensured adherence to home safety.^6^ In-home injury prevention interventions designed to increase parental recognition of household dangers may reduce the likelihood of these events. Parents, however, commonly misjudge their child’s motor skill, cognitive ability and judgment, underestimating some and overestimating others.^7^

Interventions designed to improve and assess parents’ ability to identify household hazards have relied on handouts, pamphlets, photos, and use of actual rooms set up for the purpose.^6,8,9^ An alternative tool that has received less attention is video of actual rooms presented to parents on a laptop or other portable device. The current study utilizes videos of household dangers filmed at two different heights: 5’8” (adult point of view) and 3’ (child point of view). The purpose of this study was to compare two pairs of videos (four videos total: bedroom and kitchen from both a child’s point of view (POV) and an adult’s POV) to determine whether the height at which the video was taken influenced the number of hazards parents could identify in a child’s bedroom and in a kitchen. We hypothesized that parents viewing videos taken at a child’s perspective would identify more hazards than parents viewing similar videos taken from an adult’s perspective. Ultimately, this information will be helpful to create tools and educational modules designed to educate families on how to increase safety in their households. 

## Methods 

**Study design**

This prospective study assessed parents’ ability to identify household dangers illustrated in short videos. The videos were designed to demonstrate ten household hazards, five each in an actual child’s bedroom and an actual kitchen. The videos were filmed using a head-mounted video camera worn on a 3 year old’s head (3’, child’s POV) or an adult’s head (5’8”, adult POV). The number of hazards identified by parents who viewed adult height videos were compared to the number identified by parents who viewed child’s point-of-view videos.

**Instrumentation**

Each study arm included two 3-minute videos of a typical child’s bedroom and a kitchen. For each room, the person (either a 3 year old child or an adult) wearing the camera entered the rooms, walked around, and made video contact with five distinct, staged hazards. The audio was muted.

The child’s bedroom included the following five hazards: 1) crib with pillows and stuffed animals; 2) blinds with a draped cord hanging within 30” of the floor; 3) electrical power strip on the floor; 4) open purse with a pharmaceutical vial (orange with a white lid); and 5) electric space heater. All hazards appeared real; however, the scene was set up such that the child wearing the head-mounted camera was safe. Two adults were with him, the electrical power strip and the heater were unplugged, the cord on the blinds was not attached, and the “medications” vial was empty.

The kitchen also had five hazards: 1) open cupboard with simulated cleansers and other chemicals; 2) 5-gallon bucket on the floor; 3) step stool next to a counter; 4) pan on a stove with its handle turned outward; 5) cup of steaming liquid. As with the bedroom, all hazards were simulated: bottles of cleansers and chemicals were empty and clean, the bucket was empty, the pan on the stove was empty, and the “steaming liquid” imagery was created using dry ice. Two adults were in the room at all times during filming.

**Participants**

Participants were a convenience sample of parents or guardians of patients less than 19 years old who presented to the University of New Mexico Pediatric Emergency Department between January 2017 and April 2017. Participants were approached, invited to participate, and verbally consented. In order to minimize situations which might be overly distracting to participants, parents were excluded if their children were incarcerated or in legal custody, presented with psychiatric issues, or were critically ill. Participants were randomized to view either the bedroom/kitchen pair of videos taken at an adult height, or at a child’s height. The pairs of videos were proxies for viewing the rooms from the perspective of an adult versus the perspective of a child. Based on prior research (see references above), the average parent, viewing risks from an adult height, is expected to identify <50% of the risks in each room. Each room has 5 risks, so the control group is expected to identify <5 of the 10 total hazards (50%). If the group of parents who see the dangers from a child’s height can see 60% of the hazards (3 in each room), and allowing for a standard deviation of 2, the effect size (Cohen’s d) is 0.5. To detect this effect size using alpha = 0.05, this study will have 80% power if 53 people per group are enrolled.

**Procedures**

Trained research assistants collected demographic information on a tablet device, including the participant’s gender, age, education level, and number of children living at home. Depending on randomization category, participants were asked to view the videos of the child’s bedroom and kitchen from either a child’s POV or an adult POV on a tablet device and identify hazards seen. Research assistants checked off items on a separate tablet device as they were named. Participants could not see the research assistants’ checklist. If parents named hazards not on the checklist, they were recorded but the data were not analyzed. Participants were permitted to view each video only once, and individuals whose participation was interrupted for more than five minutes were dropped from the study. Those interrupted for less than five minutes were given the option to resume participation but were not permitted to start over. After viewing both videos, participants were debriefed. Participants were shown screenshots from their respective videos showing the dangers in each room. The UNM IRB approved this study (#16-378).

**Data management and analysis**

Study data were collected and managed using REDCap electronic data capture tools hosted by the University of New Mexico. REDCap (Research Electronic Data Capture) is a secure, web-based application designed to support data capture, providing 1) an intuitive interface for validated data entry; 2) audit trails for tracking data manipulation and export procedures; 3) automated export procedures for seamless data downloads to common statistical packages; and 4) procedures for importing data from external sources.^10^

The main outcome measures were the total number of hazards identified for each room. For each correct response, participants were given one point; each room had a score range of 0-5. The primary predictor variable was the height at which the video was taken (adult vs. child). Age, education, and number of children living at home were included as control variables in multivariate analyses. 

Shapiro-Wilk normality tests of the outcome variable distribution confirmed non-normal data distribution, so non-parametric analyses were used for measures of central tendency and to test hypotheses. The Mann-Whitney U test was used to compare the number of hazards identified using the adult height versus child height videos, stratifying by room (bedroom or kitchen). Multivariable analysis employed Spearman’s rho to control for possible confounders. A Type I error rate of 0.05 was used to define significance.

## Results

The sample included 106 participants whose median age group was 31-35 years. Most participants (78.3%) had at least a high school diploma, and more than one-fourth (27.4%) had a college degree. The number of children living at home ranged from 1 (21.7%) to five or more (9.4%); the median was two. Randomization resulted in approximately equal groups of participants who viewed videos from adult (n=54; 50.9%) and child (n=52; 49.5%) point-of-view. Groups did not differ with respect to median participant age (p=0.51), education (p=0.55), or number of children living at home (p=0.64).

Table 1 shows that the proportion of parents who correctly identified individual bedroom and kitchen hazards varied by the specific hazard and, in some cases, by the height at which the video was taken. Video height was not significantly associated with identification of individual hazards in the child’s bedroom. Two kitchen hazards were identified by more of participants viewing the child height videos than the adult height videos: a bucket on the floor and a cup of steaming liquid on a counter.

**Table 1:** Hazards correctly identified by parents viewing videos of a child’s bedroom and a kitchen taken from an adult height and a child height (n=106).

Child’s Bedroom	Total n (%)	Adult Height n (%)	Child Height n (%)	p-value
Crib	44 (41.5)	23 (42.6)	21 (40.4)	0.82
Blinds	95 (89.6)	47 (87.0)	48 (92.3)	0.37
Outlets	93 (87.7)	46 (85.2)	47 (90.4)	0.42
Medications	32 (30.2)	20 (37.0)	12 (23.1)	0.12
Heater	43 (40.6)	26 (38.2)	17 (32.7)	0.10
**Kitchen**				
Open cupboard	102 (96.2)	52 (96.3)	50 (96.2)	0.97
Bucket	35 (33.0)	2 (3.7)	33 (63.5)	0.001
Step stool	57 (53.8)	32 (59.3)	25 (48.1)	0.25
Pan on stove	89 (84.0)	48 (88.9)	41 (78.8)	0.16
Steaming cup	78 (73.6)	31 (57.4)	47 (90.4)	0.001

Table 2 indicates the median total number of hazards identified in the child’s bedroom was 3 for participants watching videos taken at either adult or child height (p=0.32). Parents viewing child height videos of the kitchen identified significantly more hazards (median=4) than parents viewing adult height videos (median=3) (p=0.0001). Age was not associated with the total number of hazards identified in the child’s bedroom (p=0.16) or the kitchen (p=0.55). The number of children at home did not influence the number of hazards recognized in either room (bedroom p=0.80; kitchen p=0.93). The number of hazards identified in the child’s bedroom increased with increasing education (rho=0.31; p=0.001). This was also true for the kitchen (rho=0.21; p=0.033). Multivariable modeling to control for education demonstrated that video height remained a significant predictor of number of hazards identified in the kitchen.

**Table 2:** Median number of hazards identified, by room among parents watching videos of a child’s bedroom and a kitchen from an adult height and a child height (n=106).

	Total		Adult Height		Child Height		p-value
	Median	IQR*	Median	IQR*	Median	IQR*	
**Child’s bedroom**	3	2,4	3	2.4	3	2,3	0.32
**Kitchen**	3	3,4	3	3,4	4	3,5	0.0001

*IQR=interquartile range.

## Discussion

This study examined whether parents recognize more dangers from videos filmed from a child’s POV than from an adult’s POV. Prior research has assessed recognition of household hazards by adults but none that we know has used point-of-view (height) as a predictor. 

For example, Powell et al. compared instructing parents on injury prevention using photographs versus written injury prevention tip sheets and found no difference in recall of information, but did not use a visual evaluation.^11^Another study distinguishes characteristics of adults (parents, childcare workers, and pediatric providers) who successfully identify household injury hazards, when asked to identify dangers in simulated household rooms. Ninety-four individuals participated in identifying dangers in three simulated rooms: a living room with 35 hazards, a toddler’s bedroom with 20 hazards, and a bathroom with 15 hazards. Subjects were asked to mark hazards with stickers as they walked through the rooms. Parents identified more hazards than either daycare workers or health care providers. And, parents with a higher education level identified more hazards.^8^Mayes et al. examined caretakers’ recognition of hazards in a household. These caretakers were shown pictures of a typical household, including a bedroom, living room, kitchen, dining room and backyard. Each picture showed at least 5 hazards. On average, parents identified 52% of the 65 dangers presented. Stratifying by household income, participants whose annual income was less than $30,000 identified fewer household dangers. Younger participants identified fewer hazards than older caretakers.^9^

This study adds to the literature by adding two realistic elements to assessments of household hazards: 1) motion; and 2) comparison of child and adult viewpoints. The aim was to determine if an alternative viewpoint would help parents recognize dangers. Our data demonstrated that although video height did not influence the number of hazards identified in the bedroom, parents who observed the kitchen video taken at a child’s height identified more hazards than those viewing a video at adult height. We were able to demonstrate that subjects were able to identify more than half of the dangers, which was a higher proportion than previous studies where only photos of rooms were shown.^9^However, significantly fewer hazards were used in this study compared to Mayes et al. (10 vs 80). The demographic results were consistent with the Mayes study, in that caretakers with higher education attainment identified more dangers than those with a lower educational attainment. This was not true for the kitchen. It is unclear why education level would influence hazard identification in only one of the two rooms.

There were two kitchen dangers that were more often identified from a child’s viewpoint than from an adult’s viewpoint. The cup with simulated steam at the child’s eye level was recognized as a hazard by 90.4% of participants viewing the child POV versus 59.6% from an adult viewpoint. The other hazard was the empty 5-gallon bucket, which could be a drowning hazard (child POV 64.7% versus adult POV 3.9%). The cup with simulated steam may have been more recognizable because there was motion involved (the dry ice emission). The bucket may have been more recognizable due to effective injury prevention strategies of this hazard (e.g. warning illustrations on 5 gallon buckets) or because adults tend to spend more waking hours in the kitchen and hence more keenly aware of changes in the environment. Nonetheless, these two differences demonstrate that there is some value in showing dangers from a child’s viewpoint for educational purposes. This is consistent with the anticipatory guidance approaches of the American Academy of Pediatrics (AAP) in both Bright Futures^7^ and The Injury Prevention Program (TIPP).^12^ Bright Futures emphasizes that both developmental skills and motor skills should be considered which are often misjudged by parents; parents can overestimate cognitive abilities and judgement of their children and underestimate their motor skills.^7^ The TIPP sheets are produced by age group, reflecting probable developmental stage of the child.^12^ We believe this study demonstrates that utilizing different perspectives (POV) enhances caregiver understanding of both child perspective and developmental capabilities. 

**Limitations**

As a head-mounted camera was used to simulate point of view, motion artifact may have affected the results, by influencing recognition of objects. The sample size of 106 participants limits the study power. Each video was less than 3 minutes and we are unsure if longer videos may help or hinder parents in finding dangers in the household. The videos were limited in the number of rooms and dangers. Participants identified hazards not included in the five designated answers, but these additional hazards did not influence participants’ scores. The “other” hazards noted were diverse and included the answer “everything” which suggested that parents were on high alert and would identify anything perilous as a hazard. 

## Conclusion

The novel tool developed for this study demonstrated that teaching parents to consider household hazards from the viewpoint of the child has two potential uses. Primarily, the tool was used to evaluate parents’ current ability to identify hazards. Secondly, with the addition of the debriefing, the tool was used to teach parents to recognize potential hazards. However, we did not evaluate the efficacy of the educational component such as measuring parent knowledge acquisition or retention resulting from the debriefing. Although caregivers were the target of this study, a future study could evaluate whether the tool could be useful for others involved in home safety education, and preparedness such as Community Health Workers, health workers and other home visitation services. 

**Acknowledgments**

Thank you to Scott Oglesbee, MPH and Child Ready research assistants who enrolled participants.
